# Arteriogenic therapy based on simultaneous delivery of VEGF-A and FGF4 genes improves the recovery from acute limb ischemia

**DOI:** 10.1186/2045-824X-5-13

**Published:** 2013-07-01

**Authors:** Agnieszka Jazwa, Mateusz Tomczyk, Hevidar M Taha, Elisa Hytonen, Mateusz Stoszko, Lorena Zentilin, Mauro Giacca, Seppo Yla-Herttuala, Costanza Emanueli, Alicja Jozkowicz, Jozef Dulak

**Affiliations:** 1 Department of Medical Biotechnology, Faculty of Biochemistry, Biophysics and Biotechnology, Jagiellonian University, Gronostajowa 7, 30-387, Krakow, Poland; 2 Department of Biotechnology and Molecular Medicine, A. I. Virtanen Institute for Molecular Sciences, University of Eastern Finland, Kuopio, Finland; 3 Molecular Medicine Laboratory, International Centre for Genetic Engineering and Biotechnology (ICGEB), Trieste, Italy; 4 Laboratory of Vascular Pathology and Regeneration, School of Clinical Sciences, Regenerative Medicine Section, University of Bristol, Bristol, UK

**Keywords:** AAV, Angiogenesis, Arteriogenesis, FGF4, VEGF-A

## Abstract

**Background:**

Gene therapy stimulating the growth of blood vessels is considered for the treatment of peripheral and myocardial ischemia. Here we aimed to achieve angiogenic synergism between vascular endothelial growth factor-A (VEGF-A, VEGF) and fibroblast growth factor 4 (FGF4) in murine normoperfused and ischemic limb muscles.

**Methods:**

Adeno-associated viral vectors (AAVs) carrying β-galactosidase gene (AAV-LacZ), VEGF-A (AAV-VEGF-A) or two angiogenic genes (AAV-FGF4-IRES-VEGF-A) were injected into the normo-perfused adductor muscles of C57Bl/6 mice. Moreover, in a different experiment, mice were subjected to unilateral hindlimb ischemia by femoral artery ligation followed by intramuscular injections of AAV-LacZ, AAV-VEGF-A or AAV-FGF4-IRES-VEGF-A below the site of ligation. Post-ischemic blood flow recovery was assessed sequentially by color laser Doppler. Mice were monitored for 28 days.

**Results:**

VEGF-A delivered alone (AAV-VEGF-A) or in combination with FGF4 (AAV-FGF4-IRES-VEGF-A) increased the number of capillaries in normo-perfused hindlimbs when compared to AAV-LacZ. Simultaneous overexpression of both agents (VEGF-A and FGF4) stimulated the capillary wall remodeling in the non-ischemic model. Moreover, AAV-FGF4-IRES-VEGF-A faster restored the post-ischemic foot blood flow and decreased the incidence of toe necrosis in comparison to AAV-LacZ.

**Conclusions:**

Synergy between VEGF-A and FGF4 to produce stable and functional blood vessels may be considered a promising option in cardiovascular gene therapy.

## Introduction

Cardiovascular diseases constitute the major cause of morbidity and mortality. Gene therapy promoting therapeutic neovascularization represents a promising approach in the treatment of myocardial and peripheral ischemia and the choice of appropriate therapeutic agent is one of the important issues influencing the effectiveness of this procedure [1].

Vascular endothelial growth factor-A (VEGF-A, VEGF) is a critical regulator of angiogenesis that stimulates proliferation, migration, and proteolytic activity of endothelial cells (ECs) and is also one of the well-studied factors for therapeutic angiogenesis for post-ischemic vascular repair [2]. Numerous experiments have produced encouraging but rather short-term results. Additionally, robust increase in VEGF-A was often reported to result in the formation of an unstable and permeable vascular network undergoing regression relatively soon, due to the missed recruitment of supporting pericytes [3-6]. Moreover, exaggerated VEGF-A expression was even shown to accelerate limb amputation associated with massive muscular edema, necrosis, and disturbed regeneration [7]. The lack of expected benefits of VEGF-A therapy in humans suggests that the stimulation of therapeutic angiogenesis for the treatment of cardiac or limb ischemia with only one growth factor may be insufficient [8].

Considering that angiogenesis is a cascade of sequential events, requiring regulated interaction of various growth factors and cells to form functionally effective blood vessels, a current line of research concentrates, among others, on multiple gene transfer. VEGF-A, as the prototypical angiogenic factor, has been co-administered with agents stabilizing the newly formed blood vessels, such as angiopoetin-1 (Ang-1) [9-11], or platelet-derived growth factor-B (PDGF–B) [12-14].

Fibroblast growth factor 4 (FGF4) is a member of fibroblast growth factors family and was described as an oncogene. It is expressed during early limb development and throughout embryogenesis [15,16]. In adults, FGF4 is found primarily in some tumors [17], but also in the nervous system, intestines, and testes [18]. The therapeutic potential of this agent has been demonstrated in a rabbit model of hindlimb [19] and in a pig model of myocardial [20] ischemia. FGF4, in contrast to VEGF-A and similarly to Ang-1 and PDGF-B, seems to further stabilize the adult vasculature, making it damage- and leakage-resistant. Recently, we have shown that combined overexpression of VEGF-A and FGF4 improves cutaneous wound healing in diabetic mice, an effect associated with improved fibroblast function in the wound area [21]. However, simultaneous application of VEGF-A and FGF4 for the treatment of ischemic vascular disease has not been analyzed yet.

In this paper we described the effects of intramuscular injections of VEGF-A gene, delivered either separately or in combination with FGF4 coding sequence in adeno-associated viral vectors (AAV), on ECs proliferation, mural cell recruitment and post-ischemic blood flow recovery.

## Materials and methods

### Reagents

Dulbecco’s Modified Eagle’s Medium (DMEM) and fetal bovine serum (FBS) were from PAA (Lodz, Poland) and hVEGF-A- and hFGF4-recognizing ELISA kits were procured from R&D Systems Europe (Warszawa, Poland). pAAV-MCS and pAAV-LacZ plasmid vectors were obtained from Stratagene (Piaseczno, Poland). Proliferating cell nuclear antigen (PCNA) recognizing primary antibody (clone PC10) and Animal Research Kit (ARK) Peroxidase were procured from DAKO (Gdynia, Poland). Streptavidin Alexa Fluor 546 and Alexa Fluor 488 secondary antibodies were obtained from Invitrogen (Warszawa, Poland). Oligo(dT) primers, dNTPs, MMLV reverse transcriptase and CytoTox 96 Non-Radioactive Cytotoxicity Assay were from Promega (Gdansk, Poland). All other reagents and chemicals, unless otherwise stated, were purchased from Sigma (Poznan, Poland).

### Cell culture

Human embryonic kidney-293 (HEK-293) cells were grown in DMEM high glucose containing 10% FBS, penicillin (100 U/ml) and streptomycin (10 μg/ml). Cells were kept at standard conditions: 37°C, 5% CO2, and humidified atmosphere.

### AAV vector preparation

The cDNA for human VEGF-A isoform 165 was obtained from pSG5VEGF [22]. The cDNA for human FGF4 was subcloned by PCR with appropriate primer pairs from pCAGGS-HST plasmid (kind gift of Prof. Takahiro Ochiya, National Cancer Center Research Institute, Tokyo, Japan). Human VEGF-A and human FGF4 cDNA were subcloned to pAAV-MCS expression vector as previously described [21,23]. Serotype 2 recombinant AAV vectors, were produced using AAV Helper-Free System (Stratagene, La Jolla, CA, USA) according to the protocol previously described [21,24]. Briefly, HEK-293 cells grown at 80% confluency were co-transfected with each expression plasmid (pAAV-LacZ, pAAV-VEGF-A or pAAV-FGF4-IRES-VEGF-A) and the packaging/helper plasmid pDG (kindly provided by Dr Jurgen A. Kleinschmidt, Program of Infection and Cancer, German Cancer Research Center; Heidelberg, Germany) expressing AAV and adenovirus helper functions. Fourteen hours later fresh DMEM containing 10% FBS was given. Cells were collected 3 days after transfection in their medium and AAV were released from the cells by repeated freezing and thawing. AAV were isolated by iodixanol gradient (Optiprep) centrifugation following purification with heparin affinity chromatography (used for normo-perfused muscle transduction) or by CsCl gradient centrifugation [25] (used for ischemic muscle transduction). AAV titer (viral particles, vp) was determined by measuring the number of viral genomes by quantitative PCR using the conditions and primers previously described [21].

### AAV-mediated transduction of HEK-293 cells

HEK-293 cells were cultured at 1×10^4^ cell density in 96-well plates. The cells were exposed to different doses of AAV-LacZ ranging from 100 to 10 000 MOI (multiplicity of infection) for 72 h. After that time the transduction efficiency was determined by β-galactosidase *in situ* staining as described [21]. Additionally, conditioned media were collected for evaluation of cell death by lactate dehydrogenase (LDH) release according to vendor’s protocol. As the highest dose (10 000 MOI) did not influence cell viability, it was used for further experiments, in which the functionality of VEGF-A and FGF4 coding vectors was evaluated. These agents are secreted proteins and their level was measured in cell conditioned media with appropriate ELISA kits according to vendor’s protocol.

### Mouse hindlimb ischemia and gene transfer

All animal procedures were in accordance with the *Guide for the Care and Use of Laboratory Animals* (Directive 2010/63/EU of the European Parliament) and carried out under a license from the Ethical Committee of the Jagiellonian University. Male C57Bl/6 mice (4- to 6-months old, 25–30 g) were maintained under controlled environmental conditions (12-h light/dark cycle at approx. 23°C), and provided with standard laboratory food and water ad libitum. Anaesthetized (2,2,2-tribromoethanol, 880 mmol/kg body weight, intraperitoneally) mice received intramuscular injections (1×10^10^ viral particles in 50 μl) of AAV vectors encoding β-galactosidase gene (AAV-LacZ), human FGF4 (AAV-FGF4), human VEGF-A (AAV-VEGF-A) or both genes (AAV-FGF4-IRES-VEGF-A). In another set of experiments age-matched male C57Bl/6 mice were anaesthetized and subjected to left femoral artery ligation to induce unilateral limb ischemia. Immediately after the vessel occlusion AAV-LacZ, AAV-VEGF-A or AAV-FGF4-IRES-VEGF-A (2, 5×10^10^ viral particles in 50 μl) were injected into the ischemic adductor muscle. Since the viral preparations purified by CsCl gradient centrifugation had higher particle titers than those purified by heparin affinity chromatography, it was possible to inject higher number of AAV (AAV-LacZ, AAV-VEGF-A and AAV-FGF4-IRES-VEGF-A) into ischemic thigh muscles.

### Haemodynamic measurements

Blood flow measurements using Laser Doppler Perfusion Imager System (PIM II, Perimed) were performed on anesthetized animals with limb ischemia just after the surgery and gene transfer and weekly thereafter for 4 weeks. To determine the rate of perfusion recovery to the ischemic foot, the ischemic to non-ischemic foot blood flow ratio was calculated, as previously shown [26] and recently described [27]. Mice were sacrificed after the last Doppler reading.

### Samples collection and histological examination

All animals were euthanized *via* an anesthetic overdose. Non-ischemic (normo-perused) muscles were collected at day 7, 14, 21 and 28 from the gene transfer and divided in two parts – one part was immediately frozen and used for RNA isolation and the second part was embedded in OCT compound (Tissue-Tek), snap-frozen in liquid nitrogen and used for capillary density analyses. Additionally, a separate groups of anaesthetized ischemic and non-ischemic animals at 28 day after gene transfer, were perfused with PBS (1 minute), followed by 10% buffered formalin (10 minutes) at 100 mmHg through the abdominal aorta as previously described [28]. Both adductor muscles were harvested and placed in formalin for 48 h. After paraffin embedding, 3-μm-thick sections were cut from each sample with the muscle fibers oriented in a transverse direction, stained with hematoxylin and eosin (H&E), and examined for overall morphology and the capillary density in 25 random microscopic fields (1000x magnification) by an observer blinded to the experimental protocol [28]. The number of capillaries was additionally evaluated at day 7, 14 and 21 in the muscle sections blocked with 1% bovine serum albumin and incubated with FITC-labeled *Bandeiraea simplicifolia* I (BS-I) lectin B_4_ (FITC-lectin; dilution 1:100, Vector Laboratories). The capillary density was examined in 6 random microscopic fields (200x magnification) by an observer blinded to the experimental protocol.

### Total RNA isolation

Total RNA was isolated by lysis in 1 ml of QIAzol Total RNA Isolation Reagent using Tissue Lyzer (Qiagen). Samples were transferred to eppendorf tubes and supplied with 200 μl of chloroform. The mixture was vortexed (30 sec), incubated on ice (20 min) and centrifuged (12 000 *g*, 20 min, 4°C). Then, the aqueous phase was transferred to the new eppendorf tubes, mixed with equal amount of isopropanol, incubated overnight at −20°C, and centrifuged (10 000 *g*, 30 min, 4°C). Pellets were washed twice with ice-cold 70% ethanol and centrifuged (10 000 *g*, 10 min, 4°C). Finally, the pellets were air-dried and resuspended in 30 μl of water. Concentration and quality of RNA was determined by measuring the absorbance at 260 nm and 280 nm.

### RT-PCR analysis of gene expression

cDNA template was synthesized from 1 μg of total RNA for 1 h at 42°C using oligo-dT primers and MMLV reverse transcriptase following the manufacturer protocol. The obtained cDNA was diluted 10 times in water. Quantitative PCR (qPCR) was performed using StepOne Plus Real-Time PCR (Applied Biosystems) in a mixture containing SYBR Green PCR Master Mix (SYBR Green qPCR Kit), 50 ng of cDNA and specific primers in a total volume of 15 μl. Gene expression was evaluated with qPCR performed according to a following protocol: 95°C – 15 min; 40 cycles of 95°C - 30 seconds, 60°C - 45 seconds, 72°C - 45 seconds. The primers recognizing human VEGF-A (5′-AAGGAGGAGGGCAGAATCATCACG-3′ and 5′- CTCAGTGGGCACACACTCCAG-3′), human FGF4 (5′- CGATGAGTGCACGTTCAAGG-3′ and 5′- TTCCCATTCTTGCTCAGGGC-3′) and EF2 (5′-GCGGTCAGCACAATGGCATA and 5′-GACATCACCAAGGGTGTGCAG-3′) were used. EF2 (elongation factor 2) served as a housekeeping gene. The melting curve analysis was done using the program supplied by Applied Biosystems. Relative quantification of gene expression was calculated based on the comparative C_T_ (threshold cycle value) method (ΔC_T_ = C_T gene of interest_ – C_T housekeeping gene_).

### Immunohistochemistry

Muscle sections were deparaffinized and subjected to antigen retrieval using 0.05 M sodium citrate buffer (pH 6.0) in microwave oven. For double immunofluorescence staining tissues were washed in PBS and blocked in a solution containing 10% goat serum. After blocking tissue sections were incubated with primary antibodies recognizing proliferating cell nuclear antigen (PCNA, dilution 1:200) or with chondroitin sulfate proteoglycan (NG2, dilution 1:200, Chemicon International) followed by fluorochrome-conjugated secondary antibody (Alexa Fluor 488). The same muscle sections were stained for capillary ECs with biotinylated *Bandeiraea simplicifolia* I (BS-I) lectin B_4_ (dilution 1:100, Vector Laboratories) which was treated as primary antibody during tissue sections incubation. Lectin bound to ECs was detected with streptavidin- and fluorochrome-conjugated antibody (Streptavidin Alexa Fluor 546). All sections were mounted with DAPI (4′,6-diamidino-2-phenylindole) containing medium to visualize nuclei and proliferating ECs in the whole tissue sections (1000x magnification) by an observer blinded to the experimental protocol.

Arterioles were stained with α-smooth muscle actin (α-SMA) recognizing antibody (α-actin, clone 1A4, dilution 1:200, Sigma). Immunolabeling was visualized with the HRP-streptavidin system using an animal research kit (ARK) according to vendor’s protocol. All sections were examined for the number of small arterioles (less of 20 μm of the inner diameter) in the whole tissue sections (400x magnification) by an observer blinded to the experimental protocol [28].

### Statistical analysis

Results are expressed as mean ± SEM unless otherwise stated. One-way analysis of variance (ANOVA) followed by Bonferroni’s post-hoc test or unpaired Student’s t-test was used to evaluate the statistical significance between investigated groups. p < 0.05 was considered statistically significant.

## Results and discussion

### HEK-293 cells are efficiently transduced with recombinant AAV

**Figure 1:**
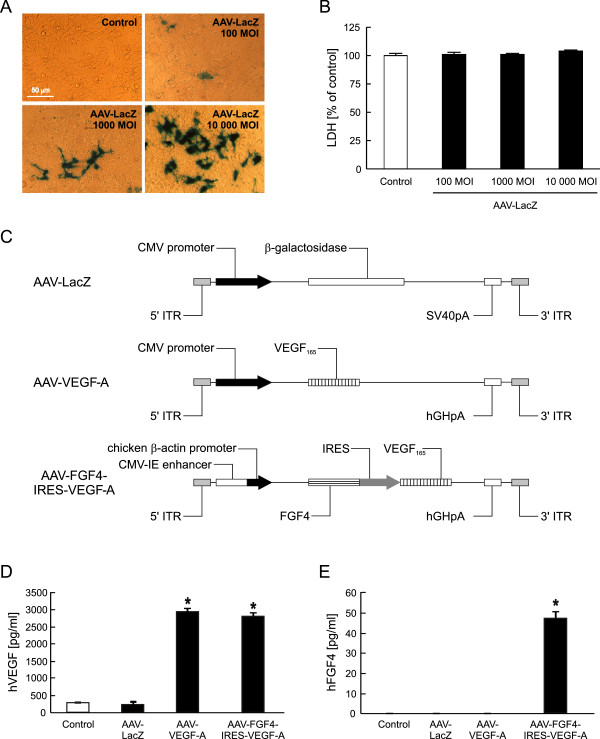
**AAV-transduced HEK-293 cells are viable and efficiently produce the protein products of the introduced transgenes. (A)** Representative pictures of β-galactosidase *in situ* staining of HEK-293 cells transduced with different doses of AAV-LacZ vector (100, 1000 and 10 000 MOI). Magnification 400x. **(B)** LDH-based cytotoxicity assay in conditioned media 72 h after transduction. **(C)** Expression cassettes of AAV vectors used in the study. **(D)** Results of ELISA determining human VEGF-A in the cell culture media. **(E)** Results of ELISA determining human FGF4 release into the cell culture media. Control stands for non-transduced cells. Representative data out of two independent experiments performed in duplicates. Values are means ± SD; *p < 0.05 vs control and AAV-LacZ.

In the present study we used AAV serotype 2 as a gene carrier because of its high natural tropism to skeletal muscle, efficient and long-term transduction, and lack of pathogenicity (reviewed in: [29]). The functionality of these gene carriers was confirmed by transduction of HEK-293 cells (Figure 1). When the cells were exposed to 100, 1000 and 10000 MOI) of AAV-LacZ, transduction efficiency was of 1.5%, 4% and 17%, respectively, as shown by β-galactosidase *in situ* staining (Figure 1A). Additionally, LDH measured in the conditioned media of transduced cells demonstrated that AAV-LacZ did not influence the cell viability at any of the tested MOI (Figure 1B). Therefore, the highest dose was used in further experiments determining the functionality of the therapeutic hVEGF-A and hFGF4 coding vectors (Figure 1C). Under normal conditions 1×10^5^ of non-infected HEK-293 cells release about 264 ± 7.5 pg/ml of VEGF-A protein within 72 h. Transduction with AAV-LacZ did not influence this production (Figure 1D). By contrast and expectedly, when either AAV-VEGF-A or AAV-FGF4-IRES-VEGF-A were added to the cells, VEGF-A release was increased by more than 10-fold (Figure 1D). Of note, the localization of VEGF-A gene after CMV promoter or IRES sequence does not seem to influence this protein production, as it was comparable after infection with monocistronic or bicistronic vector. As expected, there was no detectable level of FGF4 protein in the cell conditioned media from control HEK-293 cells (neither non-transduced nor transduced with AAV-LacZ or AAV-VEGF-A vector). At 72 h post-transduction with AAV-FGF4-IRES-VEGF-A the production of FGF4 increased up to 47 ± 2.4 pg/ml (Figure 1E).

### Both VEGF-A coding vectors transiently stimulate sprouting angiogenesis in normo-perfused skeletal muscles

**Figure 2:**
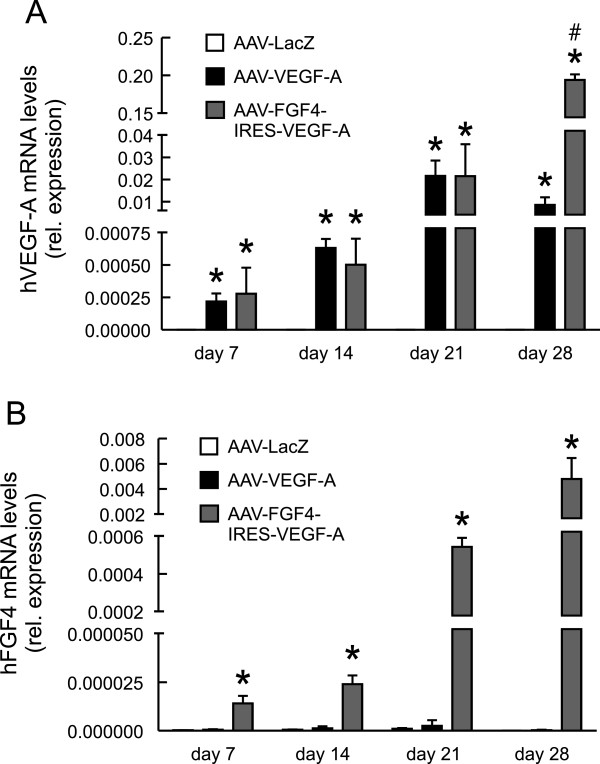
**Therapeutic transgenes are overexpressed in mouse skeletal muscles.** Quantitative PCR for **(A)** hVEGF-A and **(B)** hFGF4. EF2 served as a constitutive control gene. Values are means ± SEM (n = 3-4 per group and per time-point). * p < 0.05 vs AAV-LacZ at the appropriate time point. # p < 0.05 vs AAV-VEGF-A.

Our next aim was to check the functionality and angiogenic properties of the AAV vectors upon local intramuscular injection into the normo-perfused mouse hindlimb. We analyzed the introduced therapeutic gene expression at different time-points and found that the mRNA levels of both, human VEGF-A (Figure 2A) as well as human FGF4 (Figure 2B), increased with time. This confirms the effective transduction and expression of the locally introduced genes. Of note, we did not observe any systemic action of these agents, since their levels were undetectable in the blood serum of the animals. Accordingly with the results obtained *in vitro* in transduced HEK-293 cells (Figure 1D and E), we detected lower level of FGF4 than VEGF-A expression at all investigated time-points (Figure 2A and B). Since we delivered exactly the same dose of both, AAV-VEGF-A and AAV-FGF4-IRES-VEGF-A vectors, the level of post-transduction growth factor expression should be similar. The explanation for these differences based on the fact that both vectors contain different promoters (CMV in AAV-VEGF-A and a hybrid CMV enhancer coupled to a modified chicken β-actin promoter in AAV-FGF4-IRES-VEGF-A – Figure 1C) is rather unlikely because the later one was shown to more effectively drive the transgene expression than the regular CMV [30]. In our opinion, the difference in the post-transduction levels of hVEGF-A and hFGF4 could be explained by the fact that VEGF-A is a common protein, expressed under normal physiological conditions and necessary for maintenance of multiple cellular functions, whereas FGF4 is an agent produced in adults rather under certain pathological conditions [17]. Therefore, it is possible that more efficient FGF4 expression requires activation of some additional cellular mechanisms involved in its RNA processing and translation. Nevertheless, FGF4 was expressed and further analysis demonstrated that it was also active.

**Figure 3:**
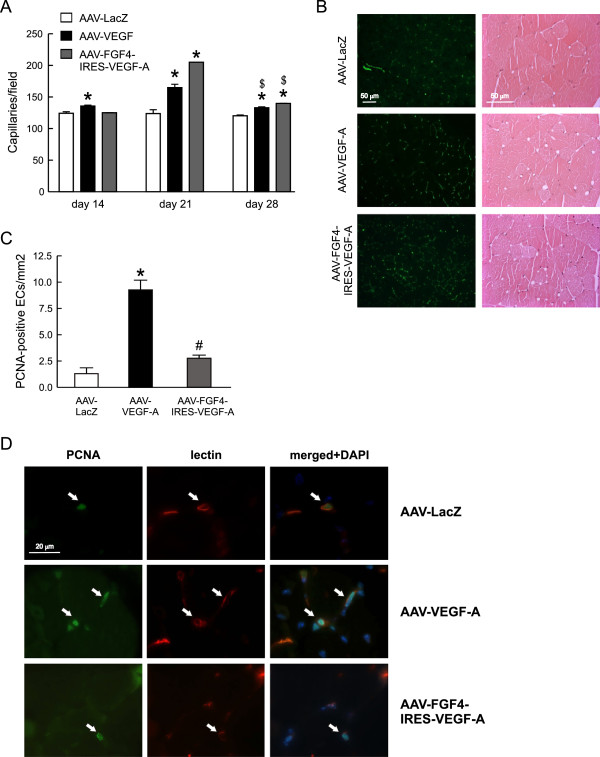
**VEGF-A gene transfer stimulates neovascularization of normo-perfused mouse adductor muscles. (A)** Capillary density quantified at different time-points after AAV-mediated muscle transduction. **(B)** Representative pictures of FITC-lectin-stained (green) capillaries 21 days after AAV-mediated gene transfer (left panel) and thigh muscle tissue histology (H&E) at day 28 following injection with different AAV (right panel). Magnification 200x and 400x, respectively. **(C)** Proliferating PCNA-positive capillary ECs in normo-perfused adductors 28 days after transduction. **(D)** Representative pictures of the double immunofluorescent staining detecting biotinylated lectin B4 *Bs* bound to ECs (red) and PCNA (green) in cells undergoing DNA synthesis. Double positive cells are indicated by arrows. DAPI was used to stain the nuclei (blue) and to confirm the nuclear localization of PCNA. Magnification 1000x. Values are means ± SEM (n = 3/group and time-point), * p < 0.05 vs AAV-LacZ. # p < 0.05 vs AAV-VEGF-A. $ p < 0.05 vs, appropriately, AAV-VEGF-A or AAV-FGF4-IRES-VEGF-A at day 21.

Histological examination of the normo-perfused adductor muscles injected with AAV-VEGF-A revealed an increased, when compared to AAV-LacZ-treated mice, number of FITC-lectin-stained capillaries at three different time-points with the highest level of induction at day 21 (Figure 3A and 3B, left panel). Accordingly, such increased vascularization followed the pattern of VEGF-A expression at these different time-points (Figure 2A). Interestingly, AAV-FGF4-IRES-VEGF-A caused even higher than AAV-VEGF-A skeletal muscle capillarization at day 21 (Figure 3A). On the other hand, despite even higher at day 28 than at day 21 VEGF expression from AAV-FGF4-IRES-VEGF-A vector (Figure 2A) the number of capillaries decreased by day 28 (Figure 3A). Therefore, in case of both AAV-VEGF-A and AAV-FGF4-IRES-VEGF-A this effect seems to be rather transient, as the number of capillaries, although still higher than in control AAV-LacZ-treated mice, dropped by day 28 (Figure 3A).

VEGF-A is a strong endothelial mitogen and its gene transfer has been widely used as a vessel growth stimulating agent in animals and humans [7,8,21,31,32]. According to different reports, however, a therapeutic strategy based on this single angiogenic factor may be inefficient and lead to the formation of morphologically and architecturally deficient and unstable blood vessels [3-6]. Recently we have shown that long-term (up to 1 year) AAV-VEGF-A gene transfer caused substantial morphological changes in skeletal muscles including aberrant vascular structures inside myofibers and skeletal muscle fibrosis [33]. Therefore, there is a need for controlling this process. In the present study, in all H&E stained muscle sections analyzed 28 days after AAV-mediated gene transfer we did not find any apparent tissue damage, abnormal vascular structures, signs of edema or hemorrhage (Figure 3B, right panel). Moreover, we did not see any significant differences in the staining against extra-vascular albumin between analyzed groups of animals (not shown), what additionally confirms the proper structure of the newly formed vessels. Obviously, we cannot exclude that this simply may be related to the relatively low VEGF-A production after AAV transduction when compared for e.g. with adenoviral vectors, other commonly used gene therapy tools.

Apparently, after performing the double immunofluorescence staining in muscle cross-sections we detected significantly higher number of proliferating PCNA-positive ECs at 28 days after local delivery of VEGF-A coding sequence in AAV-VEGF-A vector pointing at, despite decreasing growth factor expression (Figure 2A), constant activation of ECs proliferation at the site of injection (Figure 3C and D). Surprisingly, AAV-FGF4-IRES-VEGF-A bicistronic vector did not increase EC proliferation persistency in the normo-perfused thigh muscle (P = NS for comparisons vs. AAV-LacZ and p < 0.05 for comparison vs. AAV-VEGF-A) (Figure 3C and D). This suggests that initially activated by VEGF-A microvessels stop proliferating and get stabilized in the presence of FGF4.

### Simultaneous overexpression of VEGF-A and FGF4 stimulates arteriogenesis in the normo-perfused skeletal muscles

**Figure 4:**
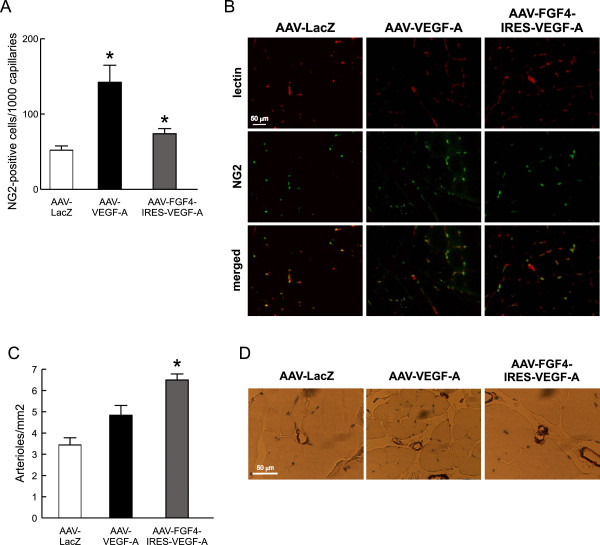
**Simultaneous administration of VEGF-A and FGF4 stimulates arteriogenesis. (A)** NG2-positive pericytes in normo-perfused skeletal muscles 28 days after AAV-mediated muscle transduction. **(B)** Representative pictures of the double immunofluorescent staining detecting biotinylated lectin B4 *Bs* bound to endothelial cells (red) and NG2 chondroitin sulfate proteoglycan on pericytes (green). Magnification 400x. **(C)** The number of arterioles significantly increased in normo-perfused skeletal muscles after AAV-FGF4-IRES-VEGF-A injection in comparison to AAV-LacZ. **(D)** Representative pictures of α-SMA-positive arterioles (arrows). Magnification 400x. Values are means ± SEM (n = 3/group), * p < 0.05 vs AAV-LacZ.

Mural cells play very important functions in the vessel wall. They not only support the vascular endothelium, but also dynamically modulate the phenotypic change from a proliferative angiogenic sprout to a proliferative quiescence of endothelium in mature microvessels [34]. In the present study we detected, in the double immunofluorescence staining of the skeletal muscle cross-sections, increased numbers of NG2-positive pericytes in the near distance of capillary ECs following AAV-FGF4-IRES-VEGF-A and AAV-VEGF-A treatment (Figure 4A and B). In fact, VEGF-A expression from adenoviral vectors was previously shown to induce relatively efficient pericyte recruitment through blood flow- and shear stress–mediated mechanisms [31,35]. Some data indicate that VEGF may act either as a vessel stabilizing or destabilizing agent depending on the dose (low doses lead to vessel maturation) [36]. AAV vectors used in the present study as gene carriers are known to provide long-term but rather low transgene expression. This might explain the increased pericyte recruitment observed after sole VEGF-A gene transfer (Figure 4A and B). Apparently, we did not observe any additive effect on these mural cells recruitment following simultaneous delivery of VEGF-A and FGF4 coding sequences and even a lower number of these cells was detected (Figure 4A and B). This prompted us to perform additional analyzes of the muscle vasculature.

The growth of arterioles in normo-perfused thigh muscles was analyzed by the immunohistochemical detection of vascular smooth muscle cells. We have observed increased number of α-SMA-positive vessels after bicistronic AAV-FGF4-IRES-VEGF-A vector administration when compared to AAV-LacZ-injected mice and to the other group receiving sole VEGF-A coding sequence (Figure 4C and D), in which arterioles were not significantly increased (p = NS vs. AAV-LacZ). Therefore, in these set of experiments FGF4 delivered in combination with VEGF-A acted as a vessel-stabilizing agent.

### Bicistronic AAV-FGF4-IRES-VEGF-A vector administration improves the post-ischemic perfusion and reduces necrosis in ischemic hindlimbs

**Figure 5:**
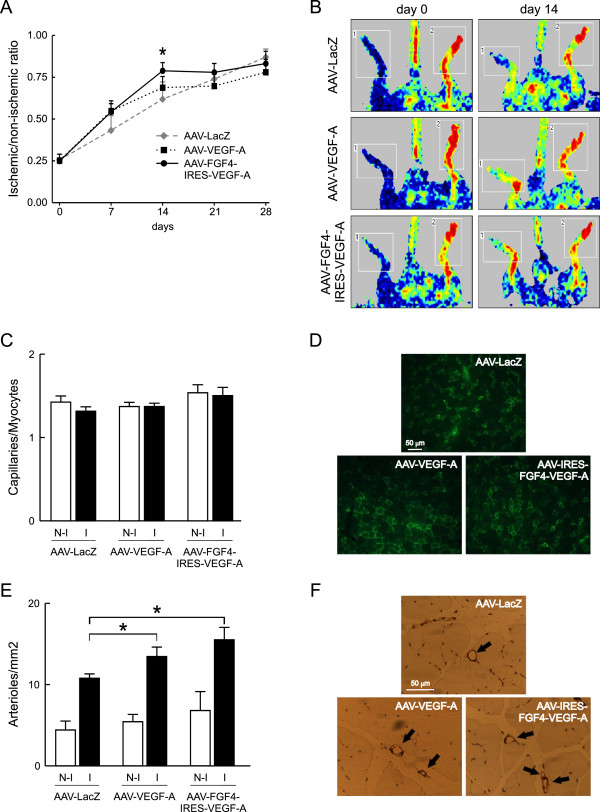
**Restoration of blood flow is faster in mouse hindlimbs injected with AAV-FGF4-IRES-VEGF-A. (A)** The time-course of the recovery of blood flow to the ischemic foot measured by Laser Doppler Flowmetry. **(B)** Typical laser doppler images of superficial blood flow in lower limbs. Squares include the area of interest (the feet) in which average perfusion was computed by the software (1 – ischemic leg, 2 – non-ischemic leg). Color scale from blue to brown indicates progressive increases in blood flow. **(C)** Total capillary density expressed as capillaries-to-myocytes ratio quantified 28 days post-surgery and AAV-mediated muscle transduction. **(D)** Representative pictures of FITC-lectin-stained (green) capillaries 28 days after femoral artery ligation and AAV-mediated gene transfer. **(E)** Arteriolar density quantified 28 days post-surgery and AAV-mediated muscle transduction. **(F)** Representative pictures of α-SMA stained arterioles (arrows) 28 days after femoral artery ligation and AAV-mediated gene transfer. Values are means ± SEM (n = 5-8/group), * p < 0.05 vs AAV-LacZ.

Increased vascularization of the normo-perfused muscles prompted us to investigate the potentially beneficial effects of the bicistronic AAV-FGF4-IRES-VEGF-A vector in comparison to AAV-LacZ and AAV-VEGF-A in a mouse model of peripheral arterial occlusive disease. Immediately after the surgery (day 0) laser doppler analysis demonstrated similar reductions of blood perfusion in all analyzed groups of animals (Figure 5A and B). Two weeks after the surgery, blood flow in ischemic hindlimbs of AAV-FGF4-IRES-VEGF-A-treated mice showed significantly better perfusion when compared to AAV-LacZ-injected animals. Post-ischemic limb perfusion following AAV-VEGF-A administration was also somewhat improved at day 7 and 14 when compared to control AAV-LacZ-treated mice, however, the differences did not reach statistical significance (Figure 5A). Importantly, the positive effect of AAV-FGF4-IRES-VEGF-A on post-ischemic blood flow recovery was associated with a markedly decreased number of necrotic toes in animals overexpressing both growth factors VEGF-A and FGF4 (0.3 ± 0.5 vs 0.8 ± 1.3 in AAV-VEGF-A and 2.0 ± 2.3 in AAV-LacZ group).

### Both AAV-VEGF-A and AAV-FGF4-IRES-VEGF-A stimulate vessel normalization in post-ischemic skeletal muscles

At day 28 post-surgery and gene transfer, the effects of reparative neovascularization response to ischemia (expressed as capillaries-to-myocytes ratio) were not observed in any of the three analyzed groups of animals (Figure 5C and D). This was most probably related to the relatively late time-point, when arterialization prevails over the sprouting angiogenesis. In fact, we have observed a significant increase in arteriolar density in response to ischemia in all analyzed groups of mice (Figure 5E and F), and it was additionally enhanced, to the similar extent though, following AAV-VEGF-A and AAV-FGF4-IRES-VEGF-A delivery (Figure 5E). Tissue ischemia by itself potently induces the growth of arterioles [37,38]. Mechanical factors derived from increased blood flow and pressure play an important role in this process. Moreover, it is important to mention that the therapeutic effect may be unclear in ischemic models because of the added complexity caused by induction of endogenous growth factors and their receptors induced by hypoxia and inflammation [19,39]. Therefore, it is reasonable to study the potential therapeutic effects in two different models – ischemic and with normal perfusion. In this study, the arteriogenic potential of AAV-FGF4-IRES-VEGF-A vector was better visible in normo-perfused skeletal muscles (Figure 4C) than in the ischemic model (Figure 5E).

The direct capability of FGFs to stimulate mesenchymal cells [40] may be considered one of the mechanisms of FGF-induced arteriogenesis. Deindl and co-workers demonstrated that arteriogenesis is associated with an increased expression and kinase activity of FGF receptor 1 in a rabbit model of hindlimb ischemia [41]. Therefore, binding and activation of this receptor may possibly be involved in the vessel wall remodeling following FGF4 administration. We have shown that production of low amounts of VEGF-A and FGF4 from AAV vectors in non-ischemic skeletal muscles results in the synergy between these two growth factors in terms of blood vessel stabilization. This effect was associated with the faster post-ischemic recovery (already at day 14) of animals injected with AAV-FGF4-IRES-VEGF-A when compared to AAV-LacZ treated controls and reduced number of necrotic events.

## Conclusions

These results demonstrate that AAV-mediated simultaneous overexpression of VEGF-A and FGF4 induces EC proliferation in a VEGF-dependent manner which can be arrested by FGF4-recruited vascular smooth muscle cells. Importantly, this combined gene transfer approach accelerates the rate of hemodynamic recovery, improves the clinical outcome of ischemic muscles and might be considered as a strategy to prevent adverse effects in critical limb ischemia.

## Abbreviations

AAV: Adeno-associated viral vector; α-SMA: α-smooth muscle actin; ECs: Endothelial cells; FGF4: Fibroblast growth factor 4; HEK-293: Human embryonic kidney cells; NG2: Chondroitin sulfate proteoglycan; VEGF-A: Vascular endothelial growth factor-A.

## Competing interests

The authors declare that they have no competing interests.

## Authors’ contributions

AJa participated in the design of the study, carried out the practical work and drafted the manuscript. MT, HMT, EH, MS and LZ participated in the practical work and discussions. MG, CE, SYH and AJo participated in design of the study and helped to draft the manuscript. JD conceived of the study, designed it and edited the manuscript. All authors read and approved the final manuscript.
